# College transition Fall 2020 and 2021: Understanding the relationship of COVID-19 experiences and psychosocial correlates with anxiety and depression

**DOI:** 10.1371/journal.pone.0287792

**Published:** 2023-07-06

**Authors:** Andrea Lourie, Susan Kennedy, Erin J. Henshaw, Drexler James

**Affiliations:** 1 Department of Psychology, Denison University, Granville, Ohio, United States of America; 2 Department of Psychology, University of Minnesota, Twin Cities, Minneapolis, MN, United States of America; Case Western Reserve University, UNITED STATES

## Abstract

Rates of mental health symptoms, particularly anxiety and depression, have increased significantly in college students in the past decade along with utilization of mental health resources. The COVID-19 pandemic created an additional source of stressors to an already challenging landscape of college transition. COVID-19 has been associated with an increase of anxiety among college students, particularly first year students, entering college in Fall 2020. The shifts in policy (e.g., federal, state, and college) accruing medical data, and vaccine availability between Fall 2020 and Fall 2021 provide an opportunity to examine the role of COVID-19 experiences in the transition to college for these two first-year student cohorts. This study examined two cohorts of first-year students, Fall 2020 and 2021, to better understand the relationship between COVID-19 experiences, psychosocial correlates, and mental health symptoms. Results suggest that for students in our Fall 2020 cohort COVID-19 experiences played a distinct role in the prediction of mental health symptoms while in Fall 2021 COVID-19 experiences did not uniquely contribute to prediction of mental health symptoms. These findings have implications for mental health interventions for first-year students transitioning to college.

## Introduction

Rates of mental health symptoms, particularly anxiety and depression, have almost doubled in college students in the past decade [1], along with concurrent utilization of mental health resources [2]. First-year students often experience separation from family or support networks, new time management demands, new academic challenges, and increased autonomy and independence. Not surprisingly, rates of mental health symptoms are consistently found to be higher in first-year college students who are undergoing the transition to college [3, 4] and experiencing multiple stressors that accompany this transition [5]. The increased rates of depression and anxiety are due, at least in part, to sleep disruption [6, 7], loneliness [8], and managing new academic demands [9]. College students report high levels of perceived stress [10], a known predictor of depression and anxiety [11, 12].

The current COVID-19 pandemic has added an additional source of stressors to the already challenging landscape of college transition. COVID-19 has been associated with an increase of anxiety among college students, particularly first year students entering college in Fall 2020 [13, 14]. Work by Lee and colleagues [15] found that more than eighty percent of college students reported pandemic-related increases in either depression, anxiety, or feelings of loneliness. Pandemic research has also identified gender differences in reports of anxiety and depression. Notably, anxiety and depression are higher for women and non-binary young adults and young adults with preexisting health challenges in comparison to young men or those with no health challenges respectively [16].

Several pandemic-related stressors disrupted the experience of first-year college students living on campuses. At many colleges, students were sent home abruptly when campuses closed due to the spread of COVID-19. Students completed their coursework remotely and in isolation. Students in their first-year transition were separated from newly developing social relationships. At home many students experienced limitations, sometimes initiated by parents, but also related to government mitigation strategies and pandemic restrictions. College students reported greater loneliness, disruptions in regular sleep and eating habits, and social isolation [14, 17]. Personal fear of illness and the fear of illness or death for family and friends was a source of significant stress for many college students [18, 19], as were concerns about continuing their education, worries about finances, and future employment opportunities [15]. Moreover, college students sometimes reported negative changes in their own preexisting health problems once the pandemic began [14]. In one review of the literature early in the pandemic Loades [20] reported that over 63 studies reported mental health impacts on previously health children in response to social isolation. Importantly, rates of anxiety and depression for adolescents and emerging adults have been predicted to increase even after the termination of COVID-19 mitigation procedures, suggesting possible long-term concerns for college student mental health [20].

Black, Indigenous, and People of Color (BIPOC) in the United States are more likely to experience systemic racism as a chronic stressor generally, and during the pandemic these factors increased college students’ risk for illness, loss, and financial disruption [21–23]. Other groups marginalized in the United States also experienced amplified risk of mental health related outcomes due to COVID-19 [24, 25]. For example, Asian, Asian American, and Pacific Islanders (AAPI) experienced heightened stressors related to anti-Asian rhetoric, hate, and violence, which increased risks for negative mental health outcomes [24–26].

By Fall 2020 students were returning to an on-campus experience. Many colleges and universities opted to provide some in-person experiences despite a spike in COVID-19 [21]. Opening was a major achievement for many small liberal arts colleges, creating opportunities for a positive on-campus experience [27]. However, the “typical” on-campus experience was necessarily changed by COVID-19 restrictions and continued pandemic-related constraints. The first-year transition was characterized by a cascade of additional potential stressors such as a heightened potential for grief, loss, change, and the need for increased adaptability because of the threats posed by COVID-19. Although college students generally were not considered at high risk for COVID-19 morbidity and mortality, research suggested that communities with college campuses were at risk for increased community rates of COVID-19 [28]. Colleges mandated mitigation policies to protect students and the communities. These policies included remote and hybrid online class learning, mask mandates, campus restrictions limiting social networking opportunities, and increased policing of student compliance behaviors [29]. In addition, campus life had additional hassles and stressors related to ongoing COVID-19 testing, threat of quarantine, being sent home for noncompliance to campus rules, and a fear of campus closure leading to a loss of the on-campus experience. Lack of social networking opportunities and social contact increased students’ feeling of loneliness. This made creating new social networks difficult particularly for first-year students leading to feelings of loneliness [30].

There were several significant shifts between Fall 2020 (Fa20) and Fall 2021 (Fa21) which again altered the landscape of the first-year transition to college during the ongoing pandemic. The promise of COVID-19 vaccines, greater scientific understanding of the virus, changing mask policies, expanded social opportunities, and a shift from a remote to in-person learning experience [21] created a unique first-year student experience. While masks and testing were still part of the first-year experience, there was a considerable loosening of campus restrictions. It is necessary to gain an understanding of how first-year college students’ mental health and pandemic experiences were related to these changes from Fa20 to Fa21.

The present study examined how COVID-19 and shifting psychosocial experiences differ between the first-year student cohorts in Fa20 and Fa21 and how these different transitional experiences are associated with student self-reported mental health symptoms and their psychosocial correlates. Specifically, we characterized how first-year students reported mental health symptoms (i.e., anxiety and depression), COVID-19 specific predictors (e.g., COVID-19 stress, fear, and attitudes towards COVID-19 restrictions) and known psychosocial correlates of the mental health outcomes (e.g., perceived stress, loneliness, social support, and sleep/somatic symptoms) were for these cohorts. We hypothesized that rates of COVID-19 fear, and COVID-19 stress would be higher for Fa20 students in comparison to Fa21 students and that women in both cohorts would have higher rates than men. Given the shifts in campus policy we also predicted more negative attitudes towards COVID-19 restrictions for Fa21 than Fa20. Finally, we examined patterns of prediction for anxiety and depression symptoms from COVID-19 experiences and psychosocial correlates separately for students in Fa20 and Fa21.

## Material and methods

### Participants

Participants in this cross-sectional study were incoming first year undergraduates (age 18 and older) at a Midwest small liberal arts college in the fall semesters of 2020 (n = 122) and 2021 (n = 58). The study was reviewed, and ethical approval granted by the Denison University Human Subjects Institutional Review Board (DU IRB FA20#1). This convenience sample was recruited through the Psychology Department Research System, electronic fliers, and fliers posted throughout the campus. Students logged into a Qualtrics form and were provided general information about the study. Interested students aged 18 years and above could then choose to read the written electronic informed consent information. If students consented to participate, they selected access to enter the study questionnaires.

During Fa20, students were mostly living residentially on campus (85.2%), while the remainder of students lived off campus and attended classes remotely. All participants lived on campus during Fa21 due to the university’s decision to return to in-person learning. Most participants were White (71%) and female (74%). Pell Grant eligibility was requested as a marker of financial need [31] (https://studentaid.gov/understand-aid/types/grants/pell); however, the small number of students reporting Pell Grants along with the number of students that did not know their Pell status made us hesitant to examine this variable in further comparisons in the study. Participant characteristics are summarized in Table 1.

**Table 1:** Participant characteristics.

	August 2020 (n = 122)		August 2021 (n = 58)	
	n	%	n	%
**Gender**				
Female	88	72	44	76
Male	31	26	12	21
Nonbinary	2	2	2	2
Did not answer	1	0	0	0
**Race/Ethnicity**				
Asian	25	21	10	17
Black/African American	9	7	2	3
Latinx, Hispanic or Spanish Origin	11	9	11	19
White	84	70	42	72
**First Generation**				
Yes	18	14	6	10
No	19	81	51	88
**Pell Eligible** ^*^				
Yes	28	23	8	14
No	65	53	34	59
IDK	28	23	16	28
**Fall 2020 Residence**				
On campus	104	85	n/a	n/a
Home	17	14	n/a	n/a
Other	1	1	n/a	n/a

Note. All students were living on campus Fall 2021.

*Federal Pell Grants are awarded to undergraduate students who display exceptional financial need.

### Measures

#### Mental health outcomes

*Anxiety*. Anxiety was measured using the 7-item Generalized Anxiety Disorder Assessment (GAD-7) [32] on a 4-point Likert scale (0 = *not at all sure* to 3 = *nearly every day*). The GAD-7 assesses the symptoms of generalized anxiety disorder. Participants report whether they have been bothered by a series of problems in the past two weeks. Sample items include: “trouble relaxing” and “feeling nervous, anxious, or on edge.” All scores were averaged. Greater values reflect greater anxiety symptoms (Cronbach’s α_**Fa20**_ = .91; α_**Fa21**_ = .93).

*Depression*. Depression was measured using the 9-item Patient Health Questionnaire (PHQ-9) [33] on a 4-point Likert scale (1 = *not at all* to 4 = *every day*). The PHQ-9 is a multipurpose instrument for screening, diagnosing, monitoring, and measuring the severity of depression. Participants respond to a series of items about how often they have been bothered by any of the following problems in the past two weeks. Sample items include: “feeling tired or having little energy” and “poor appetite or overeating.” All items were averaged. Greater values reflect greater depression symptoms and severity (Cronbach’s α_**Fa20**_ = .87; α_**Fa21**_ = .89).

#### Covid-19 experiences

*Fear of COVID-19*. Fear and anxiety related to the coronavirus was assessed using participants’ responses to a seven-item survey using a 5-point Likert scale (*1 = strongly disagree to 5 = strongly agree*) [34, 35]. Questions included “My hands become clammy when I think about the coronavirus” and “When watching news and stories about the coronavirus on social media, I become nervous or anxious.” All items were averaged, with higher values indicating greater fear of COVID-19 (Cronbach’s α_**Fa20**_ = .85; α_**Fa21**_ = .85).

#### Attitudes toward COVID-19 restrictions

Using a 6-point Likert Scale *(1 = strongly disagree to 6 strongly agree)*, participants responded to five author-generated questions asking about their attitudes related to COVID-19 restrictions. Items included “People should be able to choose whether or not to socially distance or wear masks,” “COVID-19 restrictions are harming my quality of life,” “Overall, social distancing policy responses to COVID-19 are exaggerated,” “I feel that too many restrictions have been put into place due to COVID-19,” “I find it very stressful to comply with COVID-19 restrictions.” Responses were averaged, with higher values reflecting more negative attitudes regarding COVID-19 restrictions (Cronbach’s α_**Fa20**_ = .90; α_**Fa21**_ = .90).

*COVID-19 stress*. COVID-19 related stress was measured using the *traumatic stress*, *contamination*, *danger*, and *compulsive checking* subscales of the COVID-19 Stress Scales (CSS) [36]. Broadly, the CSS measures stress- or anxiety-related responses to COVID-19. All subscales contain six items captured on a 5-point Likert scale (0 = *not at all* to 4 = *extremely*). The *traumatic stress* subscale captures traumatic stress symptoms about the pandemic (e.g., nightmares, intrusive thought; α_**Fa20**_ = .91; α_**Fa21**_ = .92). Sample item includes: “I had trouble sleeping because I worried about the virus.” The *contamination* subscale captures the fear of coming into contact with possibly contaminated objects or surfaces (α_**Fa20**_ = .91; α_**Fa21**_ = .92). Sample item includes: “I am worried that if someone coughed or sneezed near me, I would catch the virus.”

The *danger* subscale captures fear of contracting the virus (α_**Fa20**_ = .89; α_**Fa21**_ = .89). Sample item includes: “I am worried that social distancing is not enough to keep me safe from the virus.” The *compulsive checking* subscale captures compulsive checking and reassurance-seeking regarding possible pandemic-related threats (α_**Fa20**_ = .80; α_**Fa21**_ = .86). Sample item includes: “Seeking reassurance from friends or family about COVID-19.” Per each subscale, all corresponding items were averaged. Greater values represent greater stress- or anxiety-related responses to COVID-19 in each subscale.

#### Psychosocial correlates

*Loneliness*. Loneliness was measured using the 20-item revised UCLA Loneliness Scale [37]. The Loneliness Scale measures subjective feelings of loneliness as well as feelings of social isolation. Participants rate each item on a four-point Likert scale (1 = *never* to 4 = *often*). Sample items include: “I feel isolated from others” and “lack companionship”. The appropriate items are recoded, and all items are then averaged. Greater values reflect greater feelings of loneliness (Cronbach’s α_**Fa20**_ = .91; α_**Fa21**_ = .94).

*Social support*. Social support was measured using the 12-item Multidimensional Scale of Perceived Social Support (MSPSS) [38] on a 7-point Likert scale (1 = *very strongly disagree* to 7 = *very strongly agree*). The MSPSS identifies an individual’s perceived level of social support with family, friends, and significant others. Sample items include: “There is a special person who is around when I am in need” and “My friends really try to help me.” All items were averaged. Greater values reflect greater perceived social support (Cronbach’s α_**Fa20**_ = .93; α_**Fa21**_ = .94).

*Perceived stress*. Stress was measured using the 10-item Perceived Stress Scale [8] on a 5-point Likert scale (0 = *never* to 4 = *very often*). The PSS assesses the degree to which situations are appraised as stressful in the past month. Sample items include: “In the last month, how often have you felt nervous and “stressed” and “In the last month, how often have you felt that things were going your way.” The appropriate items are recoded, and all items are then averaged. Greater values reflect greater perceived stress (Cronbach’s α_**Fa20**_ = .86; α_**Fa21**_ = .84).

*Sleep/somatic symptoms*. Health symptoms were measured using the 11-item Physical Health Questionnaire (PHQ) [39]. The PHQ is a brief self-report scale of somatic symptoms including gastrointestinal problems, headaches, sleep disturbances, and respiratory illness. Sample items include: “How often have you had difficulty getting to sleep at night?”, “How many times have you had minor colds (that made you feel uncomfortable but didn’t keep you sick in bed or make you miss work)?” Participants responded to all items on a 7-point Likert scale (1 = *not at all*/0 times/1 day) to 7 = *all the time/7+times/7+days*). The appropriate items were reverse-coded then all items were averaged. Greater values reflect poorer physical health (Cronbach’s α_**Fa20**_ = .67; α_**Fa21**_ = .79).

### Procedure

The study received University IRB approval (FA20#1) prior to the start of study recruitment. Participants in the Fa20 cohort were recruited through Introductory Psychology courses, posted flyers on campus, and community announcements. Students used a QR code or email link to learn about the study and if interested were linked to the survey to complete the electronic informed consent process. Participants in the Fa20 cohort were asked to complete four waves of surveys through the online platform Qualtrics. Only the first survey wave (August 2020) is reported here. Participants in Fa20 received the choice of a $5 gift card for participating in survey 1 or research participation credits if they were enrolled in Introductory Psychology. Participants in the Fall 2021 cohort (August 2021) were all enrolled in Introductory Psychology and received research participation credit for their time completing survey. All survey information was collected through Qualtrics online survey platform.

### Data analysis

Data were analyzed using IBM SPSS v27 software. Descriptive statistics were collected to characterize the sample and to present the frequencies and percentages for the sociodemographic variables. Next, group differences in the mental health outcomes, COVID-19 experiences, and psychosocial correlates of mental health were assessed by cohort year. Given the well-developed literature on differences by gender for mental health outcomes [16] we also analyzed mental health and psychosocial outcomes by gender. The data set did not have enough students identifying as non-binary to evaluate statistical differences, so gender statistics were conducted only with categories of men and women.

Our sample was small and predominately White; for this reason, we did not focus on analyses by race/ethnicity and class year. However, given the racial inequity in experiences of COVID-19, we ran exploratory analyses to determine if there were differences between our White and Non-White students for the predictor variables in Fa20 and Fa21. Associations between all key variables were examined prior to selecting variables for a three-step model hierarchical regression analysis to determine the contribution of COVID-19 experiences and psychosocial correlates to symptoms of anxiety and depression. In the first step we included one of the known psychosocial correlates related to our outcomes, Perceived Stress for anxiety [10–12] and loneliness for depression [40]. Next, we entered COVID-19 experiences while controlling for the known predictors. In our last step we added the remaining psychosocial correlates to the model. All tests were two-tailed, and significance was set at *p <* .*05*.

## Results

Descriptive statistics for all key variables across Fa20 and Fa21 are provided in Table 2. Consistent with nationwide reporting, students endorsed high levels of both anxiety and depression symptoms. In Fa20, 36% (n = 44) of our sample reported moderate to high anxiety symptoms (i.e., scores 10 or higher) while in the Fa21, 50% (n = 29) reported scores in the moderate to high anxiety range [32]. Similarly, 44% (n = 54) of students reported depression symptoms consistent with moderate to high depression (i.e., scores 10 or higher) in the Fa20 sample compared to 55% (n = 32) in Fa21 [41].

**Table 2:** Descriptive statistics by class year 2020 and 2021.

	Class Year 2020 n = 122 2021 n = 58	M	SD	Range
**Mental Health Symptoms**				
Anxiety	2020	7.60	5.74	0–21
	2021	9.64	5.40	0–21
Depression	2020	9.16	5.84	0–22
	2021	10.85	6.01	0–24
**Psychosocial Correlates**				
Sleep/Somatic Symptoms	2020	4.16	1.24	1.21–7.29
	2021	5.18	1.32	2.71–8.43
Perceived Stress	2020	3.07	.71	1.70–4.70
	2021	3.26	.61	1.40–4.50
Fear of COVID-19	2020	2.37	.79	1.00–4.71
	2021	2.43	.87	1.00–5.00
Social Support	2020	5.36	1.14	1.58–7.00
	2021	5.34	1.25	1.08–7.00
COVID-19 Stress	2020	2.07	.68	1.00–4.58
	2021	1.87	.76	1.00–5.00
Loneliness	2020	2.55	.70	1.00–3.80
	2021	2.40	.78	1.00–3.90
Negative Attitudes towards Restrictions	2020	2.03	1.26	1.00–6.80
	2021	2.55	1.38	1.00–7.00

### Group differences by cohort year

*Mental health outcomes*. Fa21 students reported higher rates of anxiety symptoms compared to the Fa20 students. There was a trend for higher depression scores in Fa21 compared to the Fa20 students, although this comparison did not reach statistical significance.

*COVID-19 experiences and psychosocial correlates*. Students reported greater Sleep/Somatic symptoms in Fa21 than Fa20 and as hypothesized had more negative attitudes towards COVID-19 restrictions in Fa21 than Fa20, see Table 3. Exploratory analyses of the psychosocial correlates by race revealed no differences between White and Non-White students in Fa20. In contrast, in Fa21 Non-White students (M = 2.67, SD = .69) reported higher loneliness scores than White students (M = 2.21, SD = .79), t(56) = 2.25, p = .03, Cohen’s d = .75. While White students (M = 5.65, SD = 1.21) reported greater social support than Non-White students (M = 4.88, SD = 1.18) t(56) = 2.39, p = .02, Cohen’s d = 1.20.

**Table 3:** Independent samples t-test for mental health symptoms and psychosocial correlates by cohort year.

	2020 (n = 122)		2021 (n = 58)		t(df)	p
	Mean	SD	Mean	SD		
Mental Health Outcomes						
Anxiety	7.60	5.74	9.64	5.40	t(178) = 2.27	**.02**
Depression	9.13	5.84	10.85	6.01	t(178) = 1.80	.07
Psychosocial Correlates						
Sleep/Somatic Symptoms	4.16	1.24	5.18	1.32	t(178) = 5.03	**.001**
Perceived Stress	3.07	.71	3.26	.61	t(178) = 1.82	.07
Fear of COVID-19	2.37	.79	2.43	.87	t(178) = .45	.65
Social Support	5.36	1.14	534	1.25	t(178) = .12	.91
COVID-19 Stress	2.07	.68	1.87	.76	t(178) = 1.76	.08
Loneliness	2.55	.70	2.39	.78	t(178) = 1.35	.18
Negative Attitudes towards restrictions	2.03	1.26	2.55	1.38	t(178) = 2.52	**.02**

#### Group differences by gender

*Mental health outcomes*. Consistent with previous research, women reported higher rates of anxiety symptoms compared to men. The ratings of depression were marginally higher for women compared to men although the comparison did not reach statistical significance.

*COVID-19 experiences and psychosocial correlates*. There were several gender differences for the psychosocial correlates, see Table 4. Men reported more negative attitudes towards restrictions than women. Women reported more Sleep/Somatic symptoms, higher perceived stress, more fear of COVID-19, and greater social support in comparison to men.

**Table 4:** Independent samples t-test for mental health symptoms and psychosocial correlates by gender.

	Female (n = 132)		Male (n = 43)		*t(df)*	*p*
	*Mean*	*SD*	*Mean*	*SD*		
**Mental Health Outcomes**						
Anxiety	9.00	5.85	6.12	4.67	*t*(172) = 2.91	**.004**
Depression	10.12	6.13	8.33	5.12	*t*(172) = 1.71	.09
**Psychosocial Correlates**						
Sleep/Somatic Symptoms	4.77	1.31	3.71	1.08	*t*(172) = 4.70	**.001**
Perceived Stress	3.26	.68	2.74	.58	*t*(172) = 4.41	**.001**
Fear of COVID-19	2.50	.77	2.04	.82	*t*(172) = 3.30	**.001**
Social Support	5.50	1.16	5.03	1.17	*t*(172) = 2.23	**.03**
COVID-19 Stress	2.04	.70	1.87	.62	*t*(172) = ***1***.43	.154
Loneliness	2.45	.74	2.65	.71	*t*(172) = 1.56	.121
Negative Attitudes towards restrictions	2.07	1.26	2.55	1.45	*t*(172) = 2.07	**.04**

### Bivariate correlations

Bivariate correlations were evaluated for all key variables. As expected, anxiety and depression symptoms were significantly correlated with each other and our psychosocial correlates. Consistent with the literature, perceived stress was highly correlated with both anxiety and depression as were loneliness, and sleep/ somatic symptoms. The social support variable was negatively associated with both anxiety and depression symptoms, but the correlations were not robust in the individual cohorts. Unsurprisingly, Fear of COVID-19 and COVID-19 stress, which measure overlapping constructs, were also highly correlated. Correlations are reported in Table 5.

**Table 5:** Bivariate correlations for all variables (n = 180).

	1.	2.	3.	4.	5.	6.	7.	8.
**1. Anxiety**								
**2. Depression**	.74**							
3. Fear of COVID-19	.33**	.20**						
4. Negative Attitudes towards restrictions	.10*	.16*	-.09					
5. COVID-19 Stress	35**	.22**	.69**	-.07				
6. Loneliness	.35**	.45**	.08	.03	.18*			
7. Social Support	-.15*	-.23**	-.02	-.08	-.09	-.58**		
8. Perceived Stress	.70**	.63**	.21**	.12	.20**	.30**	-.07	
9. Sleep/Somatic Symptoms	.56**	.52**	.34**	.08	.26**	.13	-.05	.57**

** Correlation is significant at the .01 level (2-tailed)

* Correlation is significant at the .05 level (2-tailed)

### Regression analyses

To understand how psychosocial correlates, predict mental health symptoms in first year students and how these experiences might have differed for those entering in Fa20 versus Fa21, we examined regression models predicting anxiety and depression symptoms separately by year. We were interested in whether the patterns of prediction were different for COVID-19 variables in Fa20 and Fa21. As noted above bivariate analyses revealed multicollinearity between Fear of COVID -19 and COVID-19 Stress, so we selected only COVID-19 Stress for subsequent regression analyses. Although social support is often included in predictors for both anxiety and depression, in previous research [30] these variables were not strongly associated with the mental health outcome variables in this sample, so they were not included in the subsequent regression analyses.

*Anxiety*. Given the substantial data supporting perceived stress as a predictor of anxiety [10], we utilized a hierarchical multiple regression analyses to determine if COVID-19 experiences (i.e., COVID-19 stress and negative attitudes towards restrictions) and psychosocial correlates (i.e., Sleep/Somatic symptoms and Loneliness) would improve the prediction of anxiety after accounting for student perceived stress.

*Fall 2020*. A hierarchical multiple regression was conducted to determine if the addition of COVID-19 experiences (COVID-19 stress and negative attitudes towards restrictions) and then psychosocial correlates (Sleep/Somatic symptoms and Loneliness) improved the prediction of anxiety symptoms over and above reported perceived stress. See Table 6 for full details on each regression model. In relation to anxiety, when perceived stress was entered into the model (Model 1) there was a statistically significant, *R*^2^ = .56, *F*(1, 120) = 150.32, *p* < .001. The addition of COVID-19 experiences to the prediction of anxiety (Model 2) led to a small but statistically significant increase in *R*^2^. The full model of perceived stress, COVID-19 experiences, and psychosocial correlates to predict anxiety (Model 3) was statistically significant. Both COVID-19 Experiences and psychosocial correlates significantly contribute to the final model.

**Table 6:** Hierarchical multiple regression predicting anxiety from perceived stress, COVID-19 predictors, and psychosocial correlates Fall 2020.

	Model 1			Model 2			Model 3		
Variable	B	β	t	B	β	t	B	β	t
Constant	-10.79**			-13.10**			-14.91**		
Perceived Stress	5.99	.75	12.26**	5.37	.67	10.68**	4.56	.57	7.52**
COVID-19 Experiences									
COVID-19 Stress				1.55	.18	2.95*	1.38	.16	2.64*
Negative Attitudes towards restrictions				.50	.11	1.85	.51	.11	1.92
Psycho-Social Correlates									
Sleep/Somatic Symptoms							.35	.08	1.07
Loneliness							1.25	.15	2.41*
R2	.56			.60			.62		
F	150.32**			58.44**			37.96**		
Δ R2	.56			.04			.02		
Δ F	150.32**			6.11*			3.50*		

Note. n = 122

* p < .05

** p < .01.

*Fall 2021*. The hierarchical multiple regression was repeated for the Fa21 class year. See Table 7 for full details on each regression model. The addition of perceived stress to predict anxiety (Model 1) was statistically significant, *R*^2^ = .30, *F*(1, 56) = 24.20, *p* < .001. The addition of COVID-19 stress to the prediction of anxiety (Model 2) led to a statistically significant increase in *R*^2^ of .13, *F*(2, 54) = 6.19, *p* = .004. The full model of perceived stress, COVID-19 stress, and psychosocial correlates to predict anxiety (Model 3) was statistically significant, *R*^2^ = .55, *F*(5, 52) = 12.73. The addition of the psychosocial correlates to the prediction of anxiety (Model 3) produced a statistically significant increase in *R*^2^. Perceived stress and sleep/somatic symptoms contribute significantly to the final model, but COIVD-19 experiences do not significantly contribute to this final model.

**Table 7:** Hierarchical multiple regression predicting anxiety from perceived stress, COVID-19 predictors, and psychosocial correlates Fall 2021.

	Model 1			Model 2			Model 3		
Variable	B	β	t	B	β	t	B	β	t
Constant	-6.20*			-9.24*			-11.54*		
Perceived Stress	4.86	.55	4.92**	5.04	.57	5.54**	2.78	.31	2.67*
COVID-19 Experiences									
COVID-19 Stress				2.12	.30	2.87*	1.22	.17	1.71
Negative Attitudes towards restrictions				-.59	-.15	-1.43	-.59	-.15	-1.58
Psycho-Social Correlates									
Sleep/Somatic Symptoms							1.67	.41	3.40**
Loneliness							1.13	.16	1.72
R2	.30			.43			.55		
F	24.20**			13.69**			12.73**		
Δ R2	.30			.13			.12		
Δ F	24.20**			6.19*			6.84*		

Note. n = 58

* p < .05

** p < .01.

*Depression*. During the pandemic there has been a conceptual focus on the impact of loneliness on college student depression [30, 42]. Based on this literature we assessed the hierarchical multiple regression analyses to determine if COVID-19 stress and our psychosocial correlates (i.e., sleep/somatic symptoms, social support, and perceived stress) would improve the prediction of depression after accounting for student reported loneliness.

*Fall 2020*. A hierarchical multiple regression was conducted to determine if the addition of COVID-19 related stress, and then psychosocial correlates (sleep/somatic symptoms, and perceived stress) improved the prediction of depression over and above reported loneliness. See Table 8. for full details on each regression model. The full model of loneliness, COVID-19 stress, and psychosocial correlates to predict depression (Model 3) was statistically significant, *R*^2^ = .53, *F*(5, 116) = 26.33, *p* < .001. The addition of COIVD-19 stress to the prediction of depression (Model 2) led to a statistically significant increase in *R*^2^ of .08, *F*(2, 118) = 6.67, *p* < .001. The addition of the psychosocial correlates to the prediction of depression (Model 3) produced a statistical increase in *R*^2^. The final model includes statistically significant contributions from Loneliness, Negative attitudes toward COVID-19 restrictions and Psychosocial correlates (i.e., perceived stress and sleep/somatic symptoms).

**Table 8:** Hierarchical multiple regression predicting depression from loneliness, COVID-19 predictors, and psychosocial correlates Fall 2020.

	Model 1			Model 2			Model 3		
Variable	B	β	t	B	β	t	B	β	t
Constant	-1.30			-5.51*			-12.13**		
Loneliness	4.10**	.49	6.20**	3.71**	.45	5.75**	2.11*	.25	3.60**
COVID-19 Experiences									
COVID-19 Stress				1.59*	.19	2.38*	.20	.02	.35
Negative Attitudes towards restrictions				.94	.20	2.67*	.73	.16	2.45*
Psycho-Social Correlates									
Sleep/Somatic Symptoms							.72*	.15	1.96*
Perceived stress							3.58**	.44	5.22**
R2	.24			.32			.53		
F	38.48**			18.48**			26.33**		
Δ R2	.24			.08			.21		
Δ F	38.48**			6.67*			26.24**		

Note. n = 122

*p < .05

** p < .01.

*Fall 2021*. For the 2021 class, see Table 9 for full details on each regression model, the full model of loneliness, COVID-19 stress, and psychosocial correlates to predict depression (Model 3) was statistically significant, *R*^2^ = .53, *F*(5, 52) = 11.59, *p* < .001. The addition of COVID-19 stress to the prediction of depression (Model 2) was not statistically significant. The addition of the psychosocial correlates to the prediction of depression (Model 3) produced a statistically significant increase in *R*^2^. COVID-19 experiences did not contribute significantly to the final model. The Psychosocial correlates, specifically, sleep/somatic problems and perceived stress significantly contributed to the model.

**Table 9:** Hierarchical multiple regression predicting depression from loneliness, COVID-19 predictors, and psychosocial correlates Fall 2021.

	Model 1			Model 2			Model 3		
Variable	B	β	t	B	β	t	B	β	t
Constant	3.20			2.55			-14.10**		
Loneliness	3.19**	.41	3.40**	2.97*	.40	3.26*	2.97*	.39	3.97**
COVID-19 Experiences									
COVID-19 Stress				.76	.10	.76	.21	.256	.80
Negative Attitudes towards restrictions				-.23	-.05	-.42	-.21	-.05	-.49
Psycho-Social Correlates									
Sleep/Somatic Symptoms							1.73	.38	3.09*
Perceived Stress							2.77*	.28	2.33**
R2	.17			.19			.53		
F	11.57**			4.09*			11.59**		
Δ R2	.16			.14			.48		
Δ F	11.57**			.46			18.80**		

Note. n = 58

* p < .05

** p < .01.

## Discussion

The current study explored the different characteristics of COVID-19 experiences, mental health outcomes, and known psychosocial correlates of mental health in two first-year student cohorts entering college during the pandemic. Moderate to severe symptoms of anxiety (41%) and depression (48%) were identified in our sample. Consistent with the national average, these numbers suggest a continued need for attention to specific mental health programming for students as they adjust to the demands of college life. These students were surveyed in the early fall semester so the need to have programming in place as students begin the fall semester is clear.

Also consistent with previous research, women reported significantly higher levels of anxiety symptoms and a trend towards higher levels of depressive symptoms; however, both men and women endorsed moderate to severe symptoms of both. Intervention and prevention programming might benefit from targeting the distinct needs of men and women separately. For example, women in our sample reported more sleep/somatic issues and greater perceived stress, they also reported more social support a known buffer of poor mental health outcomes [10]. More research is necessary to target the specific intervention needs by gender. Unfortunately, we were unable to examine non-binary students due to very small sample size; this is a group at high risk for mental health issues and warrants continued research and focus for intervention [16]. Exploratory analyses in our sample showed a difference between White and Non-White students only in the Fa21 year. Although this result is exploratory, and we are not able to break down specific racial/ethnic groups, the analyses suggest more loneliness and less social support for Non-White students. Higher rates of loneliness and lower social support may result from a sustained impact of COVID-19 experiences. This is consistent with previous work suggesting that communities of color are more likely to experience ongoing mental health sequela post pandemic [22].

In addition to examining rates of mental health problems, we also examined how COVID-19 experiences and psychosocial correlates might predict both symptoms of anxiety and depression. In the 2020 cohort COVID-19 experiences played a distinct role in predicting both anxiety and depression. Although much of the variance in student anxiety, about 30–50% for anxiety, was associated with perceived stress, COVID-19 stress did contribute to prediction of anxiety while negative attitudes towards restrictions did not uniquely contribute to predicting anxiety. Similarly, perceived stress and loneliness play a significant role in predicting depression. The importance of COVID-19 experiences in predicting mental health outcomes were diminished in the 2021 cohort. Specific COVID-19 experiences no longer added to our prediction for either anxiety or depression. Sleep/somatic symptoms and perceived stress predicted anxiety while loneliness, sleep/somatic symptoms, and perceived stress predicted depression. Once these factors are considered, COVID-19 experiences do not play a big enough roll to be measured in predicting anxiety symptoms, or depression symptoms in 2021.

While direct COVID-19 experiences had a large impact on college student mental health at the beginning of the pandemic [14] our study suggests that by 2021, perhaps due to shifts in vaccines, treatments, and understanding of the virus, the importance of COVID-19 specific experiences in understanding mental health outcomes is not direct. It is possible that COVID-19 experiences were less stressful as vaccines, education, and health providers provided better information publicly. The COVID-19 health policies incorporated in many college campuses may have buffered students’ fear and anxiety of COVID-19 specific factors. Work by Perez and colleagues [19] identified only a moderate relationship between generalized anxiety and COVID-19 fear in a college sample. While COVID-19 fear may be useful in both clinical and research settings in understanding mental health needs it may be most relevant to clinical intervention when case rates are high or during surges of new variants.

While the experience of COVID-19 does not seem to be a direct factor in mental health outcomes for our 2021 cohort the lingering effects from social isolation and loneliness, lack of social support, financial insecurity, and pandemic related learning loss are still an important consideration. It is evident that there is ongoing need for mental health interventions. Effective interventions would include stress reduction programs, delivered in a variety of ways, which have been shown to reduce stress, anxiety, and depression [43, 44] and improve mental health outcomes. Sleep disruption plays a significant role in student risk for mental health issues. Sleep interventions may also improve mental health outcomes in college students [45]. Interventions that teach effective coping strategies may also improve student mental health [46].

It is possible that college campuses may need to return to more austere restrictions for COVID-19 or respond to new health threats. There is no one solution to future pandemic responses and much will depend on the nature and risk of future public health threats. Any future planning would need to balance physical health risk with mental health needs. We have a growing understanding of how to reduce the impact on mental health during subsequent COVID-19 surges or other potential public health emergencies. College students fear social isolation and potential loneliness [47] so educational awareness and the creation of practical interventions and social support for isolating students could have positive benefits reducing loneliness, negative attitudes towards restrictions, and the related mental health impacts.

COVID-19 policies that limit new students to socializing within only one small group (e.g., randomly assigned roommate) or by proximity (e.g., people on your hallway) do not provide the meaningful interactions necessary to grow social networks. Thoughtful strategies for small group in-person social opportunities are critical especially for first-year students to create meaningful relationships and a sense of belongingness to campus. These connections are known to reduce feelings of loneliness and increase perceptions of social support [48]. Even with austere restrictions finding opportunities to create small in-person group activities is critical for students’ mental health. Working with students to create authentic, safe, and meaningful interactions would increase the ability of new students to create social networks that provide social support and reduce feelings of loneliness [30].

Appropriate mental health interventions may change as waves in the pandemic fluctuate. Focus on specific health related fears should be incorporated in mental health interventions as necessary. In general, mental health interventions, particularly focused on reducing high rates of stress and increasing effective coping skills, should be made openly available to all students. Special outreach to engage students should be tailored and targeted for the needs of the most vulnerable campus populations. Small group interventions targeting specific concerns related to student identity would be an impactful way to connect new students in the college community. COVID-19 outreach should include BIPOC students, AAPI students, LGBTQ+, and Women. Mental health interventions instituted now may be preventive if austere measures are reinstituted. Dogan and colleagues [49] found rates of depression and anxiety were decreased in a sample of Turkish college students who had visited the on-campus mental health center for these disorders prior to the pandemic.

### Limitations

Our study has several limitations. The relatively small sample was recruited at a single small liberal arts college in the Midwest United States. Females comprise more than 74% of our sample and most of our participants were White (70%). The sample Fall 2021 cohort was only 58 students, about half of the students we had hoped to recruit. We recognize that the power to identify potential associations with COVID-19 in 2021 is limited and replication of these findings is needed. Clearly a larger more diverse sample would be more representative of the college student population. Finally, we did not assess the mental health of individual participants prior to this study. Prior mental health has been shown as a strong predictor of pandemic related mental health symptoms in prior research [22]. In this sample we were concerned about the validity of self-reported mental health status in our college sample. Examining the individual longitudinal impact of previous mental health symptoms with our current population would have been instructive. Future research would benefit from inclusion of prior mental health status when possible.

## Conclusions

Transitioning to college is associated with multiple stressors including academic stress, financial stress, and social stress. Currently, in the United States, students are also experiencing stress related to political polarization, a rise in racial violence, and insecurity in the economic future. While the direct contribution of COVID-19 experiences to mental health symptoms appears to have diminished, students’ reports of anxiety and depression symptoms remain high. It is likely that the ongoing and cumulative impact of pandemic isolation and loneliness continue to impact student mental health. Mental health interventions related to stress management, time management, sleep hygiene education or intervention, successful coping strategies, and experiences that support successful social integration into college life are needed to ensure positive mental health outcomes on college campuses.
